# Fisetin inhibits inflammation and induces autophagy by mediating PI3K/AKT/mTOR signaling in LPS-induced RAW264.7 cells

**DOI:** 10.29219/fnr.v65.6355

**Published:** 2021-03-25

**Authors:** Yue Sun, Hong Qin, Huihui Zhang, Xiangling Feng, Lina Yang, De-Xing Hou, Jihua Chen

**Affiliations:** 1 Xiangya School of Public Health, Central South University, Changsha, China; 2 Inspecting Agency, Shanghai Municipal Health Commission, Shanghai, China; 3 Course of Biological Science and Technology, The United Graduate School of Agricultural Sciences, Department of Food Science and Biotechnology, Faculty of Agriculture, Kagoshima University, Kagoshima, Japan

**Keywords:** fisetin, macrophage, PI3K/AKT/mTOR pathway, inflammatory response, autophagy

## Abstract

**Background:**

Fisetin, a natural potent flavonoid, has various beneficial, pharmacological activities. In this study, we investigated expression changes of the fisetin regulating genes in lipopolysaccharide (LPS)-treated RAW264.7 cells and explored the role of fisetin in inflammation and autophagy.

**Methods and results:**

Microarray analysis identified 1,071 genes that were regulated by fisetin in LPS-treated RAW264.7 cells, and these genes were mainly related to the process of immune system response. Quantitative real-time polymerase chain reaction and Bio-Plex analysis indicated that fisetin decreased the expression and secretion of several inflammatory cytokines in cells administered with LPS. Western blot analysis and immunofluorescence assay showed that fisetin decreased microtubule-associated protein 1 light-chain 3B (LC3B) and lysosome-associated membrane protein 1 (LAMP1) expression in LPS-treated cells, while the autophagy inhibitor chloroquine (CQ) could partially reverse this effect. In addition, fisetin reduced the elevated expression of p-PI3K, p-AKT and p-mTOR induced by LPS in a concentration-dependent manner.

**Conclusions:**

Fisetin diminished the expression and secretion of inflammatory cytokines and facilitated autophagosome-lysosome fusion and degradation in LPS-treated RAW264.7 cells via inhibition of the PI3K/AKT/mTOR signaling pathway. Overall, the results of this study provide new clues for the anti-inflammatory mechanism of fisetin and explain the crosstalk between autophagy and inflammation to some extent.

## Popular scientific summary

- LPS induced inflammatory response and blocked autophagosome–lysosome fusion and degradation in mouse macrophages.
- Fisetin inhibited the expression and secretion of inflammatory cytokines and facilitated autophagosome–lysosome fusion and degradation in LPS-treated RAW264.7 cells via inhibition of the PI3K/AKT/mTOR signaling pathway.

Inflammation plays an important role in host defenses against invading microorganisms and injury, and it is a significant component in the pathophysiology of many chronic diseases, such as atherosclerosis (1, 2), inflammatory bowel disease (3), neurodegenerative diseases, and rheumatoid arthritis (4, 5). A coordinated series of common effector mechanisms of inflammation lead to pathological changes in various targeted tissues, such as tissue injury, oxidative stress, remodeling of the extracellular matrix, angiogenesis and fibrosis (6).

Macrophages are important mediators in the immune response against bacterial invasion and participated significantly in the regulation of the inflammatory environment (7). These have a dual role in tissue repair, regeneration and fibrosis. On the one hand, macrophages promote tissue repair and regeneration by phagocytosing cell debris and pathogen, releasing multiple growth factors, soluble mediators, and anti-inflammatory mediators. On the other hand, macrophage dysfunction could lead to uncontrolled release of inflammatory mediators and growth factors, insufficient production of anti-inflammatory mediators, and failed communication between macrophages and other cell lineages, thereby contributing to chronic inflammation, and eventually leading to tissue fibrosis (8). Lipopolysaccharide (LPS) is an endotoxin released by Gram-negative (G-) bacteria. Generally, LPS binds to toll-like receptor expressed in macrophages and induces the secretion of inflammatory cytokines by activating multiple inflammatory signaling pathways (9, 10). It has been reported that LPS translocates to the liver, and activated macrophages play a central role in the development of alcoholic hepatitis. Hao et al. have proved that hepatic ATF4 plays a pathological role in alcohol-induced mitochondrial dysfunction and liver injury by disrupting the nuclear respiratory factor 1 (NRF1)–mitochondrial transcription factor A (TFAM) pathway (11).

Autophagy is a catabolic process in which cytoplasmic contents are delivered to lysosomes through double-membrane autophagosomes for bulk degradation to maintain cellular homeostasis. At present, more than 30 autophagy-related genes and gene products have been identified. The microtubule-associated protein 1 light chain 3B (LC3B) is one of the structural proteins of autophagosomal membranes and is analyzed as a key marker for autophagosomes (12).

Lysosome-associated membrane protein 1 (LAMP1) are major protein components of the lysosomal membrane with a large luminal domain, one transmembrane domain, and a C-terminal cytoplasmic tail. The presence of LAMP1 molecules is one of the major definitions of the lysosomal membrane (13, 14). The autophagy regulatory network is very complex, which involved multiple signaling pathways, including TORC1, Ras/PKA, Sch9, AMPK, eIF2α kinase, and so on (15). It has been proved that autophagy plays a critical role in the differentiation and development of both mammals and invertebrates (16), and functions in tumor suppression (17). There is a bidirectional communication between autophagy and inflammation. Multiple immune mediators induce or suppress autophagy, while a return to homeostasis following a robust immune response is critically dependent on autophagy (18). In fact, autophagy importantly participated in macrophage function (inflammatory response and phagocytosis) (19).

Fisetin, a natural potent flavonoid with a polyphenolic structure, belongs to a class of plant secondary metabolites and is widely found in a variety of fruits and vegetables. It has various pharmacological activities, including anti-inflammatory, anti-apoptotic, anti-mutagenic, antioxidative, anti-carcinogenic and so on (20). Several epidemiological studies have shown that high consumption of dietary flavonoids could reduce the risk of coronary heart disease (21, 22), cancer (23, 24), stroke (25), and other chronic diseases. The mechanisms of fisetin preventing and inhibiting chronic inflammation-related conditions have also been proved in cell culture and animal models (26).

Although fisetin has been shown to exert anti-inflammatory activity by suppressing JNK phosphorylation and NF-κB activation in LPS-treated RAW264.7 cells (27, 28), its effects on macroautophagy in macrophages are still unknown. In this study, we examined the genes targeted by fisetin in LPS-treated macrophages using a high-throughput technology. Based on the outcomes of microarray analysis, we attempt to explore the effect of fisetin on autophagy and the intrinsic relationship between inflammation and autophagy. In conclusion, we hope to provide further evidence for the mechanism of anti-inflammatory activity of fisetin from the perspective of autophagy.

## Materials and methods

### Reagents and antibodies

Fisetin was purchased from Selleck (Houston, TX) and dissolved in dimethyl sulfoxide (DMSO). LPS and methyl thiazolyl tetrazolium (MTT) were purchased from Sigma-Aldrich (St. Louis, MO). Dulbecco’s modified Eagle’s medium (DMEM) and fetal bovine serum (FBS) were purchased from Gibco (Grand Island, NY). Penicillin–streptomycin solution (100×) and trypsin were purchased from Gen-View (Calimesa, CA). TRIzol reagent was obtained from Thermo Fisher (St. Louis, MO).

Antibodies against the following proteins were used: PI3 kinase p110α (C73F8) rabbit mAb (#4249, CST, MA), phospho-PI3 kinase p85 (Tyr458)/p55 (Tyr199) Antibody (#4228, CST, MA), phospho-mTOR (Ser2448) (D9C2) XP® Rabbit mAb (#5536, CST, MA), LAMP1 (C54H11) Rabbit mAb (#3243, CST, MA), AKT monoclonal antibody (60203-2-Ig, Proteintech, IL), phospho-AKT (Ser473) monoclonal antibody (66444-1-Ig, Proteintech, IL), mTOR polyclonal antibody (20657-1-AP, Proteintech, IL), LC3B rabbit pAb (A7198, ABclonal, MA), α-tubulin mouse mAb (AC012, ABclonal, MA), horseradish peroxidase (HRP)-conjugated goat anti-mouse IgG (IH-0032, Dingguo, China), and HRP-conjugated goat anti-rabbit IgG (H + L) (IH-0011, Dingguo, China).

### Cell culture

RAW264.7 cell line, one mouse macrophage cell line, was obtained from the Advanced Research Center of Central South University (Changsha, China). Cells were cultured in DMEM medium supplemented with 10% FBS and penicillin–streptomycin solution at 37°C in a humidified incubator containing 5% CO_2_.

### Cell viability assay

The MTT assay was used to detect the cell viability. Cells were seeded in 96-well plates at a concentration of 5 × 10^3^ cells/well, cultured for 24 h, and subsequently treated with different concentrations of fisetin (0, 5, 10, 20, 40, 80, and 160 μM) for 24 h or exposed to LPS (40 ng/mL) for 6 h after fisetin treatment (10, 20, 30 μM ) for 30 min. Afterwards, 110 μL MTT solution (MTT powder was dissolved in DMEM at a concentration of 5 mg/mL) was added into each well, and cells were incubated for 4 h at 37°C. The supernatant was then discarded, and 150 μL DMSO solution was added. After 15 min for low-speed shaking, the absorbance was detected at 490 nm using a microplate reader (BioTek, Winooski, VT). Cell viability was expressed as a percentage of MTT reduction. Each experiment was repeated four times.

### Microarray and bioinformatic analysis

RAW264.7 cells were pretreated with or without 10 μM fisetin for 30 min, and then exposed to 40 ng/mL LPS for 6 h. Total RNA was extracted using an RNeasy Mini Kit (Qiagen, Valencia, CA) following the manufacturer’s protocol. The quantity of RNA was assessed using an Agilent 2100 bioanalyzer (Agilent, Palo Alto, CA). Total RNA (500 ng) was amplified using a low RNA input fluorescent linear amplification kit (Agilent) according to the manufacturer’s protocol. Sample cRNA was labeled with cyanine 5 (Cy5), while the universal mouse reference RNA (Agilent) was labeled with cyanine 3 (Cy3). Mouse microarrays (Agilent) containing 22,050 oligonucleotides were used for hybridization following the Agilent microarray processing protocol. Briefly, Cy3- or Cy5-labeled cRNA was mixed and incubated with a microarray slide using an *in-situ* hybridization kit. After washing and drying, microarray signals were scanned using a Agilent model G2505A microarray scanner. Initial microarray chip images were analyzed using Agilent Feature Extraction software. The cutoff criteria for differentially expressed genes (DEGs) were fold change ≥ 2.0 or ≤ 0.5 between two groups.

The Gene Ontology (GO) knowledgebase is the world’s largest source of information on the functions of genes, which classifies gene function into three categories: biological process, molecular function, and cellular component. The GO analysis of DEGs was performed using the Database for Annotation, Visualization and Integration Discovery (DAVID) software, version 6.8 (http://david.abcC.ncifcrf.gov).

### Quantitative real-time PCR

Total RNA was used to synthesize cDNA using an RNA reverse transcription kit (Thermo Fisher, Waltham, MA) following the manufacturer’s protocol. Six inflammatory genes (IL-1α, IL-1β, IL-6, IL-10, Ccl3, and GM-CSF) and β-actin were quantified by quantitative real-time polymerase chain reaction (qPCR) using a LightCycler 96 (Roche, Basel, Switzerland). Reaction mixtures (10 μL) were amplified with 45 cycles of 95°C for 10 s, 60°C for 10 s, and 72°C for 10 s. The following primers were used in this study: IL-1α, F: 5′-CCCATGATCTGGAAGAGACCA-3′, R: 5′-CAAACTTCTGCCTGACGAGC-3′; IL-1β, F: 5′-G CAGTGGTTCGAGGCCTAAT-3′, R: 5′-GCTGCTTCA GACACTTGCAC-3′; IL-6, F: 5′-CTCTCTGCAAGA GACTTCCATCC-3′, R: 5′-AAGTCTCCTCTCCGGAC TTGT-3′; IL-10, F: 5′-GGCGCTGTCATCGATTTC TC-3′, R: 5′-ATGGCCTTGTAGACACCTTGG-3′; Ccl3, F: 5′-TACAGCCGGAAGATTCCACG-3′, R: 5′-GTC AGGAAAATGACACCTGGC-3′; GM-CSF, F: 5′-CA GGGTCTACGGGGCAATTT-3′, R: 5′-ACAGTCCGT TTCCGGAGTTG-3′; β-actin, F: 5′-GATCAAGATC ATTGCTCCTCCTG-3′, R: 5′-AGGGTGTAAAACGC AGCTCA-3′. The 2^−ΔΔ Cq^ method was used to calculate the relative mRNA levels.

### Bio-Plex assay and ELISA

Cell supernatants were collected, and the concentrations of IL-1α, IL-1β, IL-6, IL-10, Ccl3 and granulocyte-macrophage colony-stimulating factor (GM-CSF) were assessed using Bio-Plex Pro Mouse Cytokine Panel (Bio-Rad, Hercules, CA). The assay was performed using a Bio-Plex machine (Bio-Plex 200 System, Bio-Rad) following the manufacturer’s protocol, and the data were analyzed with the Bio-Plex manager software. Cell supernatants were collected, and the concentrations of IL-6 and TNF-α were measured with a mouse enzyme linked immunosorbent assay (ELISA) kit (ABclonal, Boston, MA) according to the manufacturer’s instructions. The absorbance was detected at 520 nm using a microplate reader (BioTek, Winooski, VT).

### Western blot

Cells were lysed using radioimmunoprecipitation assay (RIPA) lysis buffer (Beyotime, Shanghai, China) containing 1 mM protease inhibitor cocktail (Bimake, Shanghai, China) and sonicated for 15 min using an ultrasonic cell disrupter system (SCIENTZ, Ningbo, China). The supernatants were collected by centrifugation at 10,000× g for 15 min. Proteins were separated by sodium dodecyl sulfate-polyacrylamide gel electrophoresis, and then blotted onto the polyvinylidene difluoride membranes. Subsequently, the membranes were blocked with 5% non-fat milk for 1 h at room temperature, and incubated with primary antibodies overnight at 4°C and secondary antibodies for 1 h at room temperature. Finally, target proteins were detected using a chemiluminescence imaging system (Tanon, Shanghai, China) and quantified by ImageJ software.

### Immunofluorescence assay

Cells were cultured on coverslips in 24 well plates and were sequentially treated with 4% paraformaldehyde solution (Solarbio, Beijing, China) for 40 min, 0.1% Triton-X (Solarbio, Beijing, China) for 20 min, and goat serum (Boster, Wuhan, China) for 1 h. Afterwards, cells were incubated with a mixture of LC3B and LAMP1 primary antibodies overnight at 4°C, incubated with a secondary antibody mixture for 2 h at room temperature, and stained with DAPI for 5 min. Finally, slides were mounted with antifade mounting medium (Boster, Wuhan, China) and observed under a confocal laser scanning microscope (Leica, German). Cells without primary antibodies incubation were used as a negative control.

### Statistical analysis

Except for the microarray experiment, other experiments were performed at least three times. Data were expressed as mean ± SD. Statistical analysis was performed with the unpaired Student’s *t*-test or one-way analysis of variance (ANOVA) by SPSS 18.0 (Chicago, IL), and *P* < 0.05 was considered to be statistically significant.

## Results

### Effect of fisetin on cell viability

**Fig. 1:**
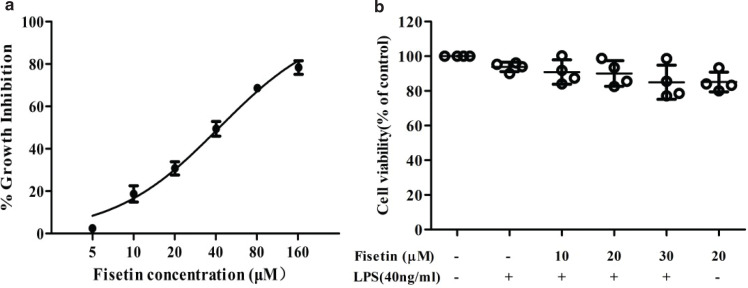
Effect of fisetin on RAW264.7 cell viability. (a) The growth inhibition rates of RAW264.7 cells treated with different concentrations of fisetin (5, 10, 20, 40, 80, and 160 μM) for 24 h; (b) The cell viabilities in the control group, the LPS (40 ng/mL) group, the fisetin (10–30 μM) and LPS (40 ng/mL) co-treatment group, and the fisetin (20 μM) group. Cells were treated with fisetin for 24 h, and then exposed to LPS for 6 h. Cell viability was detected by MTT assay, and DMSO treatment was used as a control. Data were presented as mean ± SD (*n* = 4).

To evaluate the cytotoxic effect of fisetin on RAW264.7 cells, MTT assay was performed and the cell viability was examined. Figure 1a shows the growth inhibition rates of RAW264.7 cells treated with different concentrations of fisetin (5–160 μM) for 24 h. The inhibitory effect of fisetin on cells was enhanced with the increasing concentrations of fisetin, and the IC50 value of fisetin was estimated to be 42.19 mM. Furthermore, we assessed the cell viability in the control group, the LPS (40 ng/mL) group, the fisetin (10–30 μM) and LPS (40 ng/mL) co-treatment group, and the fisetin (20 μM) group. The cell viabilities in these groups were similar without statistical significance (Fig. 1b). Hence, 40 ng/mL LPS and 10–30 μM fisetin were considered non-cytotoxic and were chosen for further study.

### Identification of genes targeted by fisetin in LPS-treated RAW264.7 cells using high-throughput techniques

**Fig. 2:**
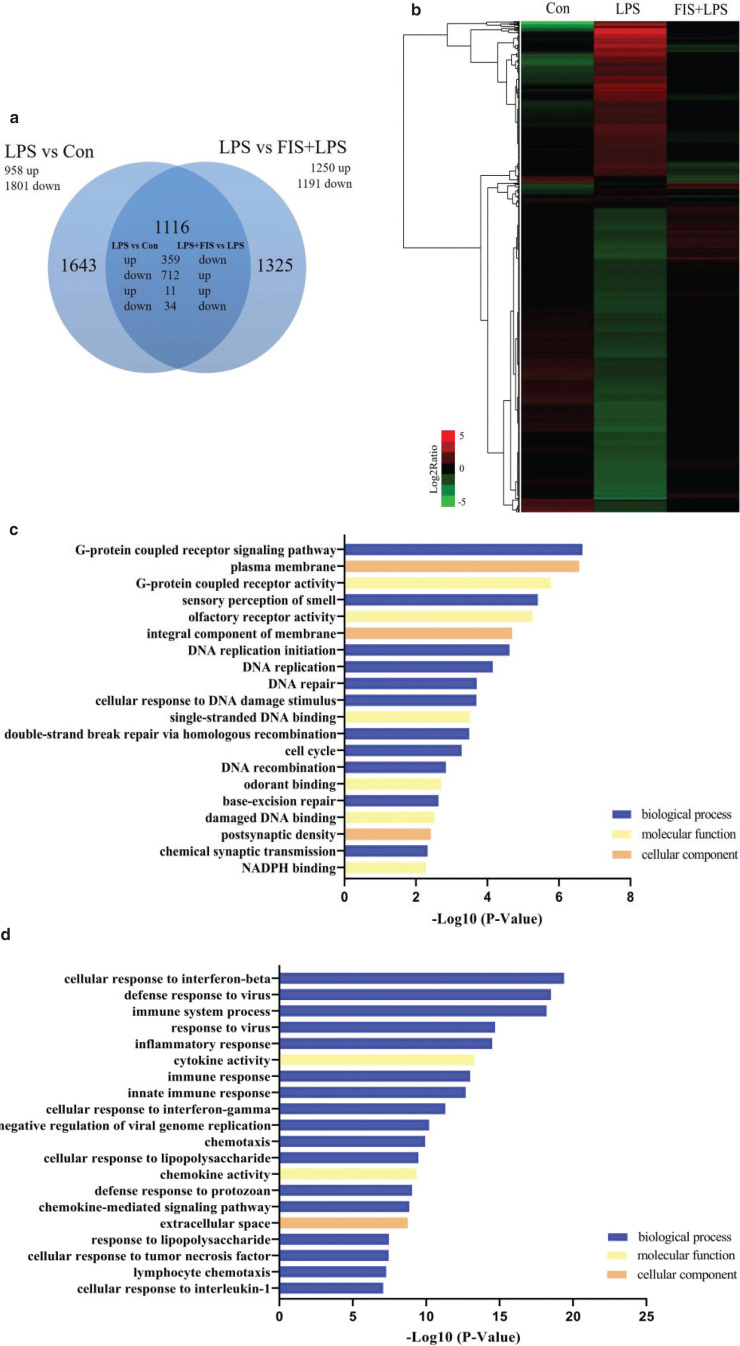
Microarray analysis of genes targeted by fisetin in LPS-treated RAW264.7 cells. (a) Venn diagram of the number of DEGs in LPS versus Con and LPS versus FIS+LPS; (b) hierarchical clustering of genes targeted by fisetin in LPS-treated RAW264.7 cells; (c) GO enrichment analysis of genes down-regulated in LPS versus Con and up-regulated in FIS+LPS versus LPS, and (d) genes up-regulated in LPS versus Con and down-regulated in FIS+LPS versus LPS. The GO terms were sorted by –Log10 of the enrichment *P*-value.

DNA microarrays were used to detect the gene expression profiles of the control group (Con), the LPS group (LPS), and the fisetin and LPS co-treatment group (FIS +LPS). Compared with the control group, 958 genes were up-regulated and 1,801 genes were down-regulated by LPS (LPS vs. Con, fold change ≥ 2.0 or ≤ 0.5). Among genes regulated by LPS, 1,071 genes were targeted by fisetin (FIS + LPS vs. LPS, fold change ≥ 2.0 or ≤ 0.5), of which 359 genes were up-regulated in LPS versus Con and down-regulated in FIS+LPS versus LPS, and 712 genes were up-regulated in LPS versus Con and down-regulated in FIS+LPS versus LPS (Fig. 2a, b and Table 1).

**Table 1:** Number of genes targeted by fisetin (FIS) in lipopolysaccharide (LPS)-treated RAW264.7 cells

Genes up-regulated by LPS and down-regulated by fisetin			Genes down-regulated by LPS and up-regulated by fisetin		
Fold change in LPS versus Con	Fold change in FIS+ LPS versus LPS	Number of genes	Fold change in LPS versus Con	Fold change in FIS+ LPS versus LPS	Number of genes
≥8	0.25< ~ ≤0.5	27	0.25< ~ ≤0.5	≥8	1
	0.125< ~ ≤0.25	21		4≤ ~ <8	55
	≤0.125	30		2≤ ~ <4	460
4≤ ~ <8	0.25 ~ ≤0.5	46	0.125< ~ ≤0.25	≥8	2
	0.125< ~ ≤0.25	32		4≤ ~ <8	42
	≤0.125	11		2≤ ~ <4	147
2≤ ~ <4	0.25< ~ ≤0.5	164	≤0.125	≥8	0
	0.125< ~ ≤0.25	27		4≤ ~ <8	2
	≤0.125	1		2≤ ~ <4	3

We then performed GO enrichment analysis to explore the function of 1,071 genes that were oppositely regulated by LPS and fisetin. For genes down-regulated in LPS versus Con and up-regulated in FIS+LPS versus LPS, terms with the highest –Log10 P-value in the biological process, molecular function and cellular component included G-protein coupled receptor signaling pathway, G-protein coupled receptor activity, and plasma membrane, respectively (Fig. 2c). For DEGs up-regulated in LPS versus Con and down-regulated in FIS+LPS versus LPS, most terms were significantly enriched in biological processes closely related to immune responses, and terms with the highest –Log10 P-value in biological process, molecular function and cellular component were cellular response to interferon-β, cytokine activity, and extracellular region, respectively (Fig. 2d).

### Fisetin inhibits gene expression and protein secretion of inflammatory cytokines in LPS-treated RAW264.7 cells

**Fig. 3:**
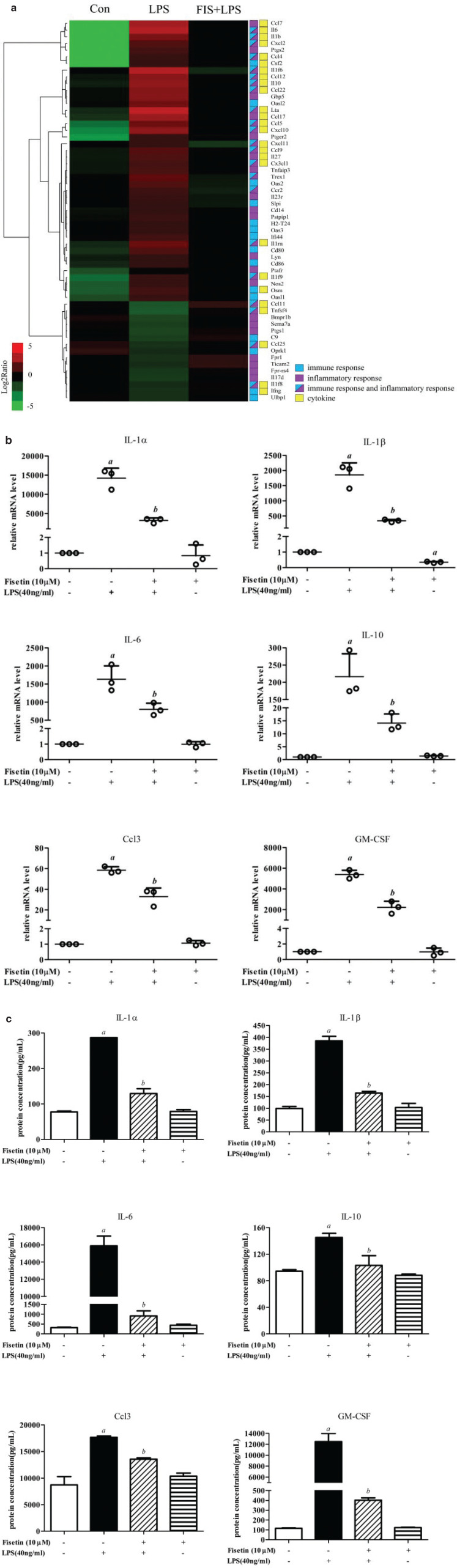
Effect of fisetin on inflammation in LPS-treated RAW264.7 cells. (a) Hierarchical clustering of DEGs related to inflammatory response or immune response. Log2 ratios were calculated based on log2 transformed output and input signals. Data were median center across gene, and Euclidean distance and the complete linkage method were used for clustering; (b) the mRNA levels of IL-1a, IL-1β, IL-6, IL-10, Ccl3 and GM-CSF were detected by qPCR; and (c) secreted proteins of IL-1a, IL-1β, IL-6, IL-10, Ccl3 and GM-CSF were detected by Bio-plex. Data are represented as mean ± SD (*n* = 3). *a* < 0.05 versus control group; *b* < 0.05 versus LPS group.

To explore the anti-inflammatory mechanisms of fisetin, we screened the genes targeted by fisetin, which were related to inflammatory response or immune response. Fisetin decreased the expression of multiple inflammatory cytokines, including interleukin family members and chemokines (Fig. 3a).

To validate the above results, we tested several inflammatory cytokines by q-PCR and Bio-plex. The mRNA levels of IL-1a, IL-1β, IL-6, IL-10, Ccl3, and GM-CSF increased after LPS exposure, and fisetin could effectively decrease the expression of these genes (Fig. 3b). Generally, cytokines are secreted into the extracellular region to exert the biological effects. Thus, we measured the protein levels of these inflammatory factors from the supernatants, and results were consistent with the microarray analysis and q-PCR (Fig. 3c). These results suggested that fisetin could inhibit the gene expression and protein secretion of multiple inflammatory cytokines, thereby exerting anti-inflammatory effects.

### Fisetin facilitates autophagosome-lysosome fusion and degradation in LPS-treated RAW264.7 cells

**Fig. 4:**
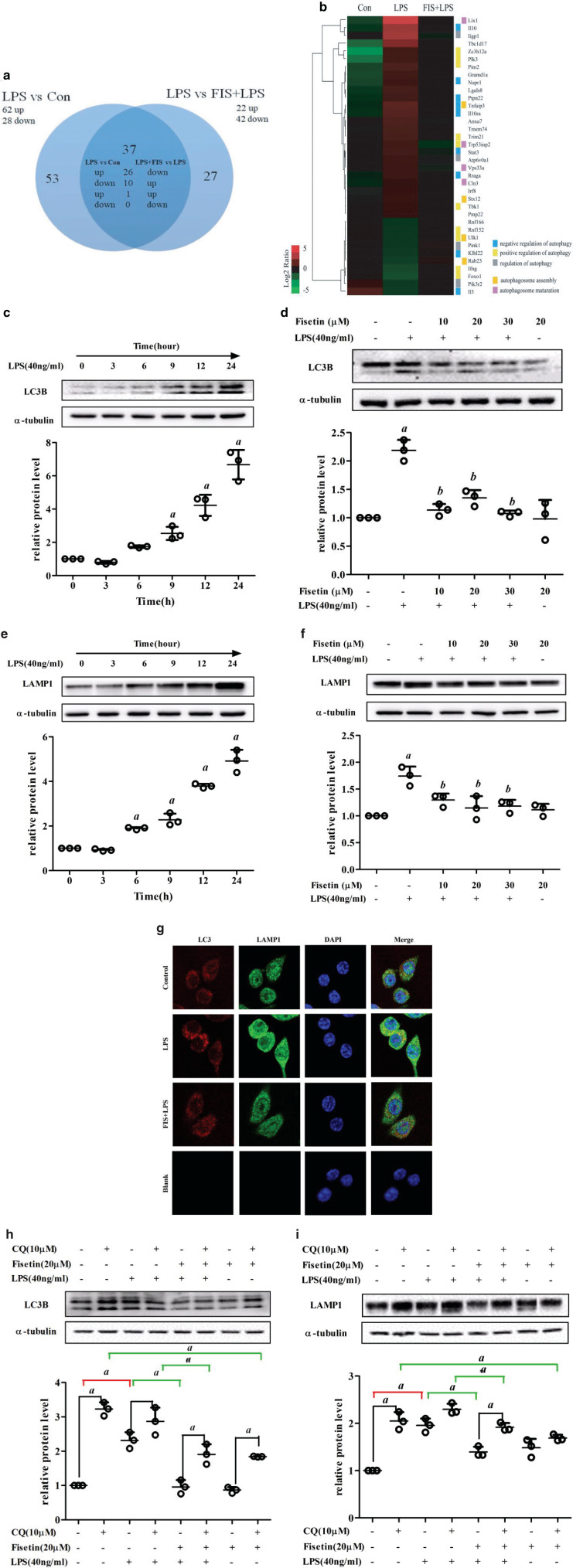
Effect of fisetin on autophagy in LPS-treated RAW264.7 cells. (a) Venn diagram of the DEGs annotated as the terms associated with autophagy. (b) Hierarchical clustering of the DEGs associated with autophagy. Log2 ratio was calculated based on log2 transformed output and input signals. Data were median center across gene, and Euclidean distance and the complete linkage method were used for clustering. (c) LC3B and (e) LAMP1 protein levels in cells exposed to LPS for different time periods (0, 3, 6, 9, 12, and 24 h); (d) LC3B and (f) LAMP1 protein levels in the control group, the LPS (40 ng/mL) group, the fisetin (10, 20 and 30 μM) and LPS (40 ng/mL) co-treatment group, and the fisetin (20 μM) group. *a* < 0.05 versus control group; *b* < 0.05 versus LPS group; (g) immunofluorescence of LC3 and LAMP1 in the control group (Control), the LPS group (LPS), the fisetin (20 μM) and LPS co-treatment group (FIS+LPS), and the negative control group (Blank); (h) LC3B and (i) LAMP1 protein levels in cells pretreated with CQ. *a* < 0.05. Data are represented as mean ± SD (*n* = 3).

As part of the cellular defense system, autophagy plays an important role in the immune response. We screened the genes targeted by fisetin that were associated with autophagy (Fig. 4a). A total of 36 genes were oppositely regulated by LPS and fisetin, and these genes mainly participated in the regulation of autophagy, autophagosome assembly, and maturation (Fig. 4b). Then we investigated the transcriptional changes of macroautophagy molecular markers. Notably, SQSTM1 decreased and Map1lc3b increased in LPS versus Con, which indicated that autophagy flux might be inhibited by LPS (Table 2).

**Table 2:** Fold changes in lipopolysaccharide (LPS) versus control group (Con) and fisetin (FIS)+LPS versus LPS for the macroautophagy molecular markers according to microarray analysis

Associated process	Gene name	Protein name	Fold change	
			LPS versus Con	FIS+LPS versus LPS
The Unc-51-like kinase (ULK) complex	ULK1	Serine/threonine-protein kinase ULK1	0.57	1.64
	ULK2	Serine/threonine-protein kinase ULK2	1.04	0.90
	ATG13	Autophagy-related protein 13	1.15	0.76
	Rb1cc1	RB1-inducible coiled-coil protein 1	1.42	0.90
The PI3K complex	BECN1	Beclin-1	1.21	1.20
	ATG14	Beclin 1-associated autophagy-related key regulator	0.67	1.75
ATG8/LC3 conjugation system	Map1lc3b	Microtubule-associated proteins 1A/1B light chain 3B	0.69	1.45
	Map1lc3a	Microtubule-associated proteins 1A/1B light chain 3A	1.28	0.95
	Gabarap	Gamma-aminobutyric acid receptor-associated protein	1.08	0.93
	Gabarapl1	Gamma-aminobutyric acid receptor-associated protein-like 1	1.43	1.23
	Gabarapl2	Gamma-aminobutyric acid receptor-associated protein-like 2	1.15	0.98
	Atg4b	Cysteine protease ATG4B	1.16	0.95
	Atg4a	Cysteine protease ATG4A	1.14	0.78
	Atg4d	Cysteine protease ATG4D	1.01	1.10
	Atg7	Ubiquitin-like modifier-activating enzyme ATG7	1.12	0.75
	Atg3	Ubiquitin-like-conjugating enzyme ATG3	1.74	1.16
ATG12 conjugation system	Atg12	Ubiquitin-like protein ATG12	1.16	1.64
	Atg7	Ubiquitin-like modifier-activating enzyme ATG7	1.12	0.75
	Atg10	Ubiquitin-like-conjugating enzyme ATG10	0.51	1.09
	Atg5	Autophagy protein 5	0.90	1.00
	Atg16l1	Autophagy-related protein 16-1	1.12	1.39
Lysosome	LAMP1	Lysosome-associated membrane glycoprotein 1	0.95	1.11
	LAMP2	Lysosome-associated membrane glycoprotein 2	1.14	1.08
Autophagy substrates	SQSTM1	Ubiquitin-binding protein p62	5.06	0.85

To clarify the effects of fisetin and LPS on autophagy, we detected autophagy marker molecules LC3B and LAMP1 by Western blot analysis. We first examined LC3B protein levels of cells exposed to LPS for different time periods (0, 3, 6, 9, 12, and 24 h). It increased in a time-dependent manner and showed significant differences after LPS exposure for 9 h (Fig. 4c). Different concentrations of fisetin could significantly inhibit LC3B expression in LPS-treated cells (Fig. 4d). LAMP1, an important marker protein of lysosome, also increased in a time-dependent manner and increased significantly after LPS exposure for 6 h (Fig. 4e). Treatment of different concentrations of fisetin decreased LAMP1 expression in LPS-treated cells (Fig. 4f). Similarly, immunofluorescence assay showed that the expressions of LC3B and LAMP were increased in the LPS group, while fisetin decreased the elevated levels of LC3B and LAMP1 induced by LPS (Fig. 4g).

To verify whether autophagy was inhibited or stimulated, cells were pretreated with chloroquine (CQ), an autophagy inhibitor that could effectively inhibit lysosomal digestion. As shown in Fig. 4h and i, CQ remarkably increased LC3B and LAMP1 accumulation. For cells without CQ pretreatment, LC3B and LAMP1 increased in the LPS group compared with the control group, and decreased by 0.57 times and 0.29 times after fisetin treatment, respectively. For cells with CQ pretreatment, LC3B and LAMP1 in the LPS group had no significant change, and decreased by about 0.33 times and 0.17 times after fisetin treatment, respectively. These results suggested that LPS blocked the fusion and degradation of autophagosome-lysosome, and treatment of fisetin could recover this process to some extent.

### Fisetin inhibits the activation of PI3K/AKT/mTOR signaling pathway in LPS-treated RAW264.7 cells

**Fig. 5:**
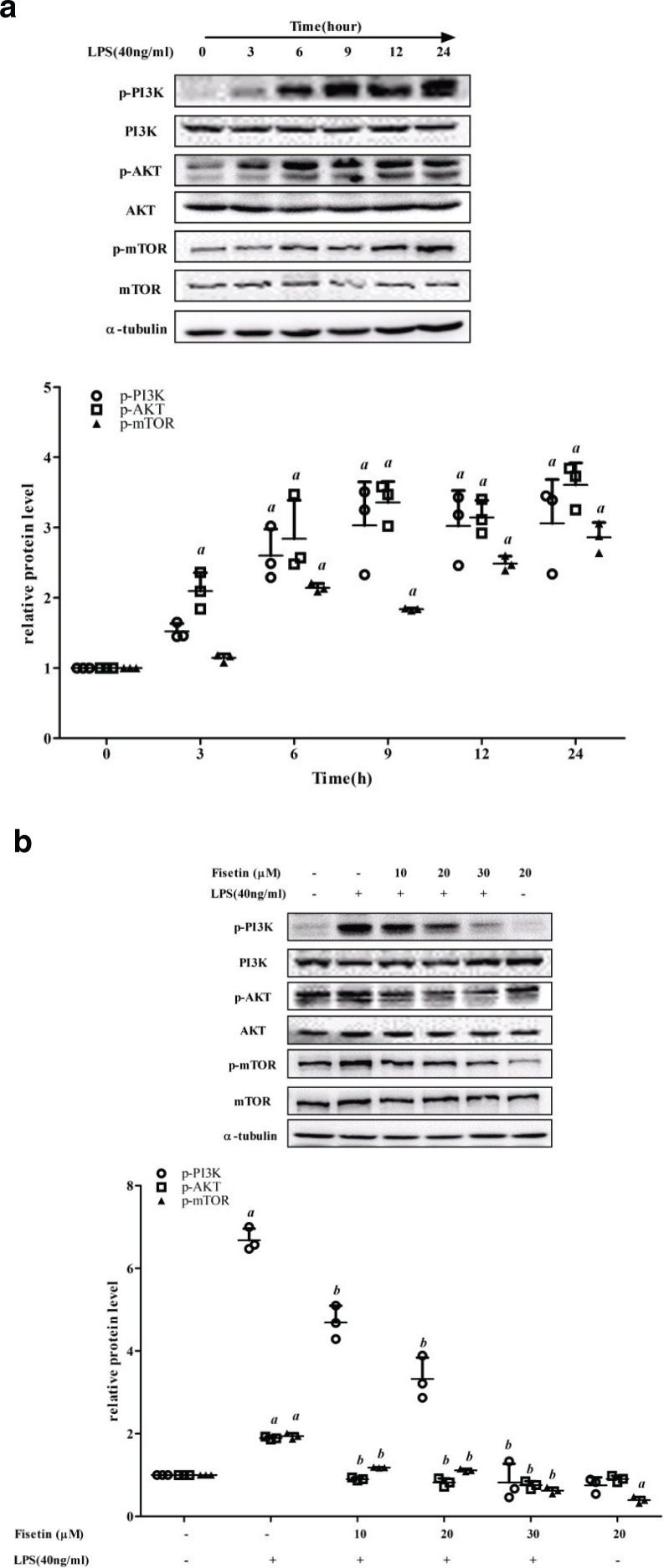
Effect of fisetin on the PI3K/AKT/mTOR signaling pathway. (a) The protein levels of p-PI3K, p-AKT and p-mTOR in cells exposed to LPS for different time periods (0, 3, 6, 9, 12, and 24 h). (b) The protein levels of p-PI3K, p-AKT and p-mTOR levels in the control group, the LPS (40 ng/mL) group, the fisetin (10, 20 and 30 μM) and LPS (40 ng/mL) co-treatment group, and the fisetin (20 μM) group. Data are represented as mean ± SD (*n* = 3). *a* < 0.05 versus control group; *b* < 0.05 versus LPS group.

To explore the possible mechanisms that fisetin facilitated autophagy in LPS-treated RAW264.7 cells, we examined the critical proteins of PI3K/AKT/mTOR signaling pathway. The protein levels of p-PI3K, p-AKT, and p-mTOR increased in a time-dependent manner in cells exposed to LPS for different time periods, and the total protein level of PI3K, AKT, and mTOR showed no significant differences (Fig. 5a). Treatment of different concentrations of fisetin decreased the protein levels of p-PI3K, p-AKT, and p-mTOR in LPS-treated cells (Fig. 5b). These results indicated that fisetin promoted autophagy by inhibiting the PI3K/AKT/mTOR signaling pathway.

### Effect of CQ on the anti-inflammatory property of fisetin

**Fig. 6:**
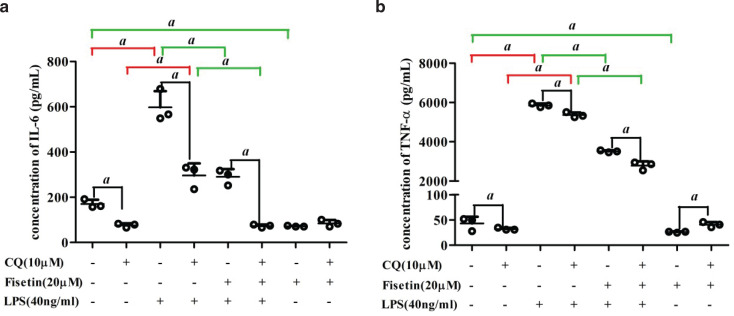
Effect of CQ on the anti-inflammatory property of fisetin. Secreted proteins of (a) IL-6 and (b) TNF-α in cells pretreated with CQ. Data are represented as mean ± SD (*n* = 3). *a* < 0.05.

To explore the correlation between autophagy activation and anti-inflammatory response induced by fisetin, interleukin-6 (IL-6) and tumor necrosis factor-alpha (TNF-α) were measured in the presence or absence of CQ. As shown in Fig. 6, for cells without CQ pretreatment, secretory IL-6 and TNF-α levels increased in the LPS group, while these decreased by about 0.51 and 0.40 times after fisetin treatment, respectively. For cells with CQ pretreatment, secretory IL-6 and TNF-α levels increased in the LPS group, while these decreased by 0.75 and 0.48 times after fisetin treatment, respectively. Compared with cells without CQ pretreatment, IL-6 in the control group, the LPS group, and the fisetin and LPS co-treatment group decreased by 0.55, 0.51, and 0.75 times in cells with CQ pretreatment, respectively. Similarly, levels of TNF-α had decreased by 0.26, 0.08, and 0.15 times in the control group, LPS group, and fisetin and LPS co-treatment group, respectively. For the fisetin group, levels of TNF-α increased in CQ-pretreated cells compared with those without CQ pretreatment, while levels of IL-6 did not change significantly.

## Discussion

Inflammation is a natural process that protects the host from tissue damage and infections, which contributes to the restoration and maintenance of tissue homeostasis. However, persistent inflammation can lead to tissue damage and dysfunction, and underlies the pathogenesis of various chronic diseases. Fisetin could inhibit or reverse the adverse inflammatory response, and is used to prevent or treat several diseases associated with persistent inflammation.

In this study, RAW 264.7 macrophages were exposed to LPS, which is considered as a classic model in inflammation research (29). Microarray analysis was performed to identify the fisetin targeting genes in LPS-treated RAW264.7 cells. We identified the DEGs in LPS versus control and FIS+LPS versus LPS, respectively. The overlapped DEGs with an opposite trend were considered as genes targeted by fisetin in LPS-treated RAW264.7 cells. GO enrichment analysis results indicated the DEGs were significantly involved in the biological process of immune system response. For DEGs that were up-regulated by LPS and down-regulated by fisetin, they were significantly enriched in terms associated with cellular response to cytokine (biological process), cytokine activity (molecular function), and extracellular space (cellular component). The results of q-PCR and bio-plex showed that fisetin decreased mRNA transcription and protein secretion of several inflammatory cytokines (IL-1a, IL-1β, IL-6, IL-10, Ccl3, and GM-CSF). These findings were consistent with several previous studies *in vitro* and *in vivo* that fisetin inhibited the production of pro-inflammatory mediators through suppression of NF-κB, JNK, and other immune-related signaling pathways in LPS-treated macrophages (28, 30, 31), and exerted anti-inflammatory activity in LPS-induced colitis (32), acute lung injury (33), central nervous system-insult (34), and acute otitis (35). It is worth noting that LPS up-regulated and fisetin down-regulated the mRNA and protein levels of IL-10, a well-known anti-inflammatory cytokine that plays a crucial role in preventing inflammatory and autoimmune pathologies.

However, aberrant expression of IL-10 can not only enhance inflammatory response to microbial challenge but also lead to development of inflammatory bowel disease and a number of autoimmune diseases (36).

Autophagy is an evolutionarily conserved metabolic process that is responsible for the lysosomal degradation of microorganisms, damaged organelles, and misfolded protein. Macroautophagy is the most typical form of autophagy. It begins with the formation of phagocytic assembly sites (PAS) and the assembly of the Unc-51-like kinase (ULK) complex, which initiates the formation of autophagosomes. In the nucleation phase, the ULK complex interacts with the class III PI3K complex to form phagocytic vesicles. The phagocytic vesicle membrane then expands to form an autophagosome. Finally, the autophagosome fuses with a lysosome and exposes cargo to the lysosomal enzymes, leading to degradation of the content. A growing number of evidences reveal that autophagy significantly participates in the innate and adaptive immunity. First, autophagy works synergistically with pattern-recognition receptors, and acts as both a regulator and an effector of PRR signaling; second, autophagy plays a significant role in the presentation of extracellular microbial antigens on major histocompatibility complex (MHC) class II in dendritic cells and the maintenance of T lymphocytes homeostasis; third, autophagy can capture and eliminate intracellular microbes; finally, autophagy positively or negatively regulates the production of multiple inflammatory factors in some specific mechanisms (19, 37, 38). In addition, it has been reported that several autophagy loci are associated with genetic predispositions for chronic inflammatory disorders and autoimmune diseases (39). The ability of fisetin to induce autophagic cell death in cancer cells has been reported in recent years. Suh et al. found that fisetin functioned as a dual inhibitor of mTORC1/2 signaling and induced autophagic cell death in PC-3 prostate cancer cells (40). Another study revealed that fisetin induced transient autophagy in response to ER stress via an AMPK-independent pathway in melanoma cells (41). Fisetin was reported to up-regulate the expression of SIRT1 and activate SIRT1 mediated deacetylation, which further decreases mTOR function and thereby induces the autophagy process (42). Fisetin can decrease Pb-induced neuroinflammation and neurodegeneration in brains by regulating the Adenosine 5'-monophosphate-activated protein kinase (AMPK)/silence information adjustment factor 2-related enzyme 1 (SIRT1) and autophagy pathway (43). Singh et al. found that the administration of fisetin significantly up-regulated the expression of autophagy genes, such as Beclin-1 and Atg-3, in the brain of induced as well as naturally aged rats (44).

In recent years, the knowledge base and new technologies for autophagy research have been continuously studied and expanded. LC3B, a widely accepted marker for autophagic activity, is associated with autophagosome development and maturation, and is used to monitor autophagic activity (45). It is worth noting that autophagy is a dynamic process and the accumulation of autophagosomes/LC3B is not necessarily equivalent to autophagy activation. In many cases, the accumulation of autophagosomes/LC3B is due to the obstruction in trafficking of autophagosomes to lysosomes without a concomitant change in autophagosome biogenesis. LAMP1 is mainly distributed on lysosome membranes and is routinely used as a lysosome marker (46). CQ has been reported to inhibit the activity of the degradative enzymes by inhibiting lysosomal acidification (47). Previous studies have demonstrated that CQ acts as an autophagy late-stage inhibitor, effectively blocking lysosomal digestion and leading to the accumulation of autophagosomes (48, 49). This study found that LC3B and LAMP1 increased in a time-dependent manner in cells exposed to LPS, suggesting that the number of autophagosomes and lysosomes increased. After inhibiting lysosomal degradation by CQ, LC3B and LAMP1 levels were comparable in the control group and the LPS group, which indicated that the increase of autophagosomes was due to the blockade in autophagosome–lysosome fusion and degradation rather than increased production. Xia et al. proved that LPS (20 ng/mL) inhibited the expression of ATP6V0D2 in bone marrow-derived macrophages, which was a key component of V-ATPase and could promote autophagosome–lysosome membrane fusion (50). However, Tan et al. found that LPS (100 μg/mL) induced podocyte injury by inhibiting the early stage of autophagy with the decreased expression of LC3 and Beclin1 and the increased expression of P62 (51). Owing to the differences in cell type and LPS intervention concentration, we observed that LPS (40 ng/mL) inhibited the late stage of autophagy and led to the increased protein levels of LC3 and LAMP1 in RAW264.7 cells. Fisetin treatment decreased the number of autophagosomes and lysosomes in LPS-treated cells. The decrease of LC3B and LAMP1 in cells without CQ pretreatment was higher than that in cells with CQ pretreatment, which suggested that fisetin facilitated the autophagosome–lysosome fusion and degradation in LPS-treated cells, resulting in decreased autophagosomes and lysosomes. Notably, for CQ-pretreated cells, the increase of LC3B and LAMP1 in the fisetin group was lower than that in the control group, which indicated that fisetin might improve the inhibition of autophagosome–lysosome fusion and degradation by CQ. Jia et al. proved that fisetin-treated human pancreatic cancer PANC-1 cells show increased autophagy, which is mediated by p8 through the p53/PKC-α pathway, and combination with autophagy inhibitors can significantly strengthen the effect of fisetin (52).

mTOR, the mammalian target of rapamycin, is a pivotal negative regulatory axis of autophagy and a downstream target of the PI3K and PKA pathways. Numerous natural products have been proved to suppress cancer cells by targeting the PI3K/AKT/mTOR-mediated autophagy (53). Wang et al. found a natural source of flavonoid, sotetsuflavone, which could induce autophagy by blocking PI3K/AKT/mTOR pathways in NSCLC cells (54). Besides, the PI3K/AmKT/mTOR signaling pathway is involved in the regulation of inflammation, which is usually related to the activation of TLRs/NF-κB, cytokine receptor and tyrosine kinase receptor signals (55). Utsugi et al. found that PI3K p110β positively regulated IL-12 production through the JNK1-dependent pathway in human macrophages and dendritic cells (56). Furthermore, salidroside exerted a pronounced cardioprotective effect in rats subjected to LPS possibly through the inhibition of iNOS, COX-2, NF-κB and PI3K/Akt/mTOR pathway (57). In this study, exposure of LPS could trigger phosphorylation of PI3K, AKT, and mTOR, and fisetin could effectively inhibit this process, which suggested that PI3K/Akt/mTOR was an effective pathway for fisetin to exert anti-inflammatory effects and increase autophagic activities.

Although the defects in autophagy are involved in several inflammatory diseases, the role of autophagy in inflammatory cytokine production remains unclear. In this study, IL-6 and TNF-α in the control group, the LPS group, and the fisetin and LPS co-treatment group decreased after CQ pretreatment. There are several speculations for this result. First, autophagy inhibits the production of inflammatory cytokines. Li et al. found that autophagy inhibitor 3-methyladenine (3-MA) attenuated the sepsis symptoms, as well as IL-6 and TNF-α production in a lethal model of murine endotoxemia and polymicrobial sepsis, while autophagy-enhancer rapamycin increased the inflammatory damages. Further research revealed that 3-MA and rapamycin might interfere with the TLR4 signaling pathway to regulate the TNF-α production in LPS-activated macrophages (58). Second, the role of autophagy in the secretion of proinflammatory factors involves more unconventional secretory pathways, rather than conventional biosynthetic pathways. It was reported that autophagy had a positive contribution to the biogenesis and secretion of the proinflammatory cytokine IL-1β via an export pathway that depends on Atg5, inflammasome, at least one of the two mammalian Golgi reassembly stacking protein (GRASP) paralogues, GRASP55 and Rab8a (59). Pu et al. demonstrated that the loss of Atg7 led to an increased production of IL-1β and enhanced inflammasome activation, but IL-6 and TNF-α levels were not changed (60). Third, in addition to being an autophagy inhibitor, CQ has been proved to be an effective anti-inflammatory drug for rheumatic diseases (61). It could reduce the deaths induced by LPS in mice through inhibition of HMGB1 release and NF-κB-mediated inflammatory pathways (62). However, we found that TNF-α levels increased after CQ treatment in the fisetin group. The decrease of IL-6 and TNF-α levels was lower in the LPS group than that in the control group, and was higher in the fisetin and LPS co-treatment group than that in the LPS group. These results suggested that the inhibition or activation of autophagy was associated with the pro-/anti-inflammation effects. Specifically, fisetin exerts anti-inflammatory effects by increasing autophagy. This inference requires confirmation in further study.

## Conclusions

This research study identified 1,071 genes regulated by fisetin in LPS-treated RAW264.7 cells using a high-throughput technology. Based on the microarray analysis, we proved that fisetin inhibited the expression and secretion of inflammatory cytokines and facilitated autophagosome–lysosome fusion and degradation in LPS-treated RAW264.7 cells via the inhibition of PI3K/AKT/mTOR signaling pathway. The findings of this study suggested that fisetin was an important anti-inflammatory bioactive compound and deserved intensive scientific exploration for the prevention and treatment of inflammation-related diseases.

## Data availability

Data used to support the findings of this study are available from the corresponding author (Jihua Chen) upon reasonable request.

## Conflict of interest and funding

The authors declare that there is no conflict of interest regarding the publication of this article. The research study was supported by the National Natural Science Foundation of China (No. 81472972).
